# Genetic interplay between human longevity and metabolic pathways — a large‐scale eQTL study

**DOI:** 10.1111/acel.12598

**Published:** 2017-04-19

**Authors:** Robert Häsler, Geetha Venkatesh, Qihua Tan, Friederike Flachsbart, Anupam Sinha, Philip Rosenstiel, Wolfgang Lieb, Stefan Schreiber, Kaare Christensen, Lene Christiansen, Almut Nebel

**Affiliations:** ^1^ Institute of Clinical Molecular Biology Kiel University 24105 Kiel Germany; ^2^ The Danish Twin Registry Unit of Epidemiology, Biostatistics and Biodemography University of Southern Denmark 5000 Odense Denmark; ^3^ Department of Clinical Genetics Odense University Hospital 5000 Odense Denmark; ^4^ Institute of Epidemiology Kiel University 24105 Kiel Germany; ^5^ Department of Clinical Biochemistry and Pharmacology Odense University Hospital 5000 Odense Denmark

**Keywords:** functional genomics, human, longevity, RNA‐ sequencing, transcriptome

## Abstract

Human longevity is a complex phenotype influenced by genetic and environmental components. Unraveling the contribution of genetic vs. nongenetic factors to longevity is a challenging task. Here, we conducted a large‐scale RNA‐sequencing‐based expression quantitative trait loci study (eQTL) with subsequent heritability analysis. The investigation was performed on blood samples from 244 individuals from Germany and Denmark, representing various age groups including long‐lived subjects up to the age of 104 years. Our eQTL‐based approach revealed for the first time that human longevity is associated with a depletion of metabolic pathways in a genotype‐dependent and independent manner. Further analyses indicated that 20% of the differentially expressed genes are influenced by genetic variants in *cis*. The subsequent study of twins showed that the transcriptional activity of a third of the differentially regulated genes is heritable. These findings suggest that longevity‐associated biological processes such as altered metabolism are, to a certain extent, also the driving force of longevity rather than just a consequence of old age.

## Introduction

Human longevity is likely to be influenced by multiple genetic and environmental factors as well as by chance (Martin *et al*., 2007). In contrast to aging, which is a continuous process occurring in all individuals, longevity is achieved only by a very small proportion of a birth cohort. The genetic component to this rare phenotype has been estimated at ~40% in long‐lived individuals (LLI) who survive beyond 85 years (Murabito *et al*., 2012). Interestingly, many of the elderly who attain such an extreme age also tend to be healthier for a longer period of their overall lifetime than their peers who died decades earlier. They achieve this extension of ‘healthspan’ by postponing the onset of major age‐related diseases and the beginning of functional decline (Andersen *et al*., 2012). In addition, LLI often show beneficial profiles for some metabolic parameters such as lipid and lipoprotein particle profiles (Barzilai *et al*., 2003). On the genetic level, nonagenarians and centenarians have been hypothesized to harbor specific alleles with protective effects, so‐called longevity variants, that may buffer or counteract the numerous disease variants they carry (Bergman *et al*., 2007; Beekman *et al*., 2010; Sebastiani *et al*., 2012). However, so far, variation in only three loci, the *APOE* gene (Schächter *et al*., 1994; Rea *et al*., 2015), the *FOXO3A* gene in the insulin‐IGF1 pathway (Willcox *et al*., 2008; Flachsbart *et al*., 2009; Soerensen *et al*., 2010), and a region of unknown function on chromosome 5q33.3 (Deelen *et al*., 2014), has been reported to influence survival beyond 90 years of age in various populations. Many more genes are assumed to play a role in human longevity, but they have remained undetected as yet despite large‐scale genome‐wide efforts (Deelen *et al*., 2011, 2014; Nebel *et al*., 2011; Beekman *et al*., 2013). As the genetic approaches have thus far provided only limited information about the determinants and molecular mechanisms underlying longevity, several studies have focused on the molecular functional aspects of the phenotype by analyzing the miRNA, epigenome, and transcriptome profiles of LLI (Rodwell *et al*., 2004; Bollati *et al*., 2009; Harries *et al*., 2011; ElSharawy *et al*., 2012; Passtoors *et al*., 2012). Yet many other studies focused primarily on aging mechanisms rather than on longevity (Van den Akker *et al*., 2014; Peters *et al*., 2015). The transcriptome of an individual reflects the influence of both genetic variation and the environment. A majority of transcriptome studies in longevity research use a cross‐sectional design (Rodwell *et al*., 2004; Harries *et al*., 2011; Passtoors *et al*., 2012), in which data of LLI are compared with that of younger participants who were born generations later. However, such a setup is not suitable to distinguish between the changes in gene expression that predispose to longevity and those that result from it (Deelen *et al*., 2013). To partly overcome this limitation, we performed in this study RNA‐sequencing‐based transcriptome profiling in blood samples of 244 LLI and younger controls and subsequently employed an expression quantitative trait loci (eQTL) analysis to investigate the association between mRNA expression levels and single‐nucleotide polymorphisms (SNPs). As the SNPs involved in the eQTLs are constitutional, they cannot be a consequence of longevity but represent one of its underlying factors. Taken together, integrating genotype information with high‐resolution transcriptome data from this sample does not only help elucidate functional mechanisms associated with longevity, but also provides for the first time the opportunity to assess the genetic contribution to it.

## Results

### Differential gene expression between LLI and CI

**Figure 1:**
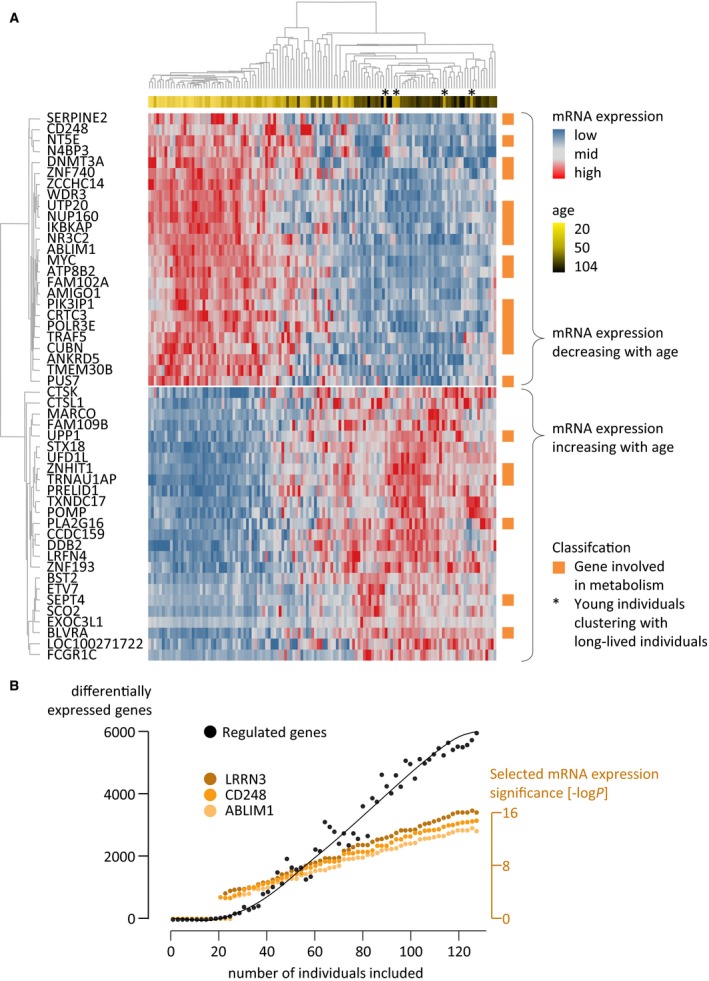
Genes differentially expressed with chronological age. (A) Hierarchical clustering of the top 50 genes, exhibiting the strongest correlation with chronological age. Each column represents an individual, while each row represents a transcript that is labeled with the corresponding gene symbol. Column dendrograms display similarities between samples and row dendrograms display transcript similarities. The orange column on the far right, which classifies genes by their involvement in metabolism, illustrates the downregulation of metabolism‐associated transcripts with age. For the top ten mRNA transcripts with positive or negative correlation with chronological age, please refer to Fig. S4 (Supporting information). (B) Differentially expressed genes identified vs. individuals included in the study setup, exemplified for the German cohort (max. *n* = 128 individuals). Randomly selected individuals were added to the analysis to estimate the resulting number of significantly differentially expressed genes (adjusted *P*‐value ≤ 0.001). For better visualization, a curve fit was added. In addition, three previously published genes (*LRRN4*,*CD248*, and *ABLIM1*) and the strongest pathway signal (GO‐term) are displayed with their significances (−log_10_ *P*‐value).

**Figure 2:**
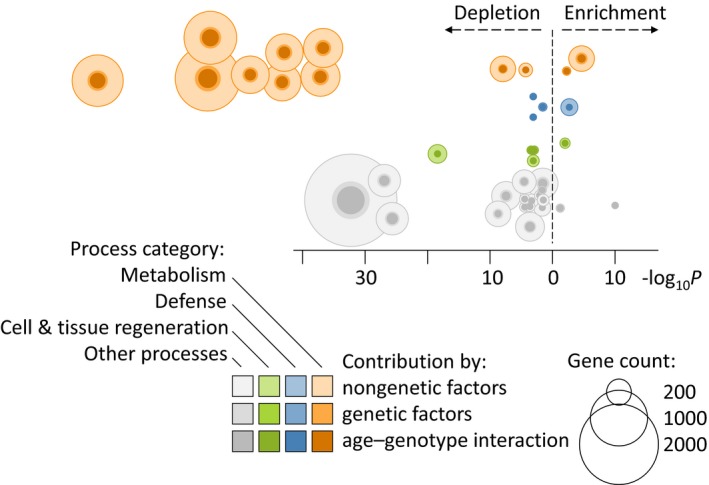
**Biological processes in longevity are controlled by genetic and nongenetic factors. For better readability, only processes with at least 150 genes are displayed. A strong depletion of processes associated with metabolism (orange) is the most prominent finding, while the effects on defense (blue) and cell and tissue regeneration (green) are less dominant. Processes that are not part of these three categories are displayed in grey. The contribution of age–genotype interaction (dark shading, inner circle) represents a part of the genetic contribution (** ***cis*** **‐** **eQTL** **, middle circle), while the remaining effects (environment, epigenetics etc.) are labeled nongenetic contribution (light shading, outer circle). The** ***y*** **‐axis is arranged by process category, while the** ***x*** **‐axis illustrates the degree of enrichment or depletion of the individual processes (−log**_**10**_ ***P*). The ten depleted metabolism‐associated processes are (orange, top left section, from left to right): cellular macromolecule metabolic process, regulation of metabolic process, macromolecule metabolic process, nucleobase‐containing compound metabolic process, cellular aromatic compound metabolic process, heterocycle metabolic process, organic cyclic compound metabolic process, cellular nitrogen compound metabolic process, protein metabolic process, and phosphorus metabolic process.**

The transcriptome analysis of 55 LLI and 73 CI (CI: control individuals, German samples) identified 19 483 of 24 934 genes analyzed as expressed in blood. Of these, 6214 were significantly differentially expressed (Benjamini‐Hochberg corrected *P*‐value ≤ 0.001; false discovery rate FDR ≤ 0.1%) between LLI and CI including 3525 downregulated and 2689 upregulated genes in LLI (Fig. 1, Table S1, Supporting information, Appendix S1: Supplemental raw data, Supporting information). Of those 6214 differentially expressed genes, 139 exhibited a high correlation (*r* ≥ 0.8) with individual blood cell types (Palmer *et al*., 2006) and were excluded from further analysis to control for altered cell compositions due to age. Subjecting the remaining 6075 differentially expressed genes to a gene ontology analysis resulted in 107 significant biological processes. We found that eight of the ten most significantly enriched or depleted biological processes were functionally linked to metabolism, which is very unlikely to have occurred by chance (*P* = 3.51 × 10^−5^; Fischer's exact test). In addition, a larger number of downregulated genes than expected by chance were associated with these eight metabolic processes, which represents the most prominent finding of the study. Most gene ontology categories were relatively broad, for example, such as cellular macromolecule metabolic process, nucleobase‐containing compound metabolic process, heterocycle metabolic process, or cellular aromatic compound metabolic process (Table S2, Supporting information); however, upon closer inspection, many of them seemed to point to nucleotide metabolism. Modulation was also found in processes functionally linked to defense as well as to cell and tissue regeneration, yet to a much lesser extent than in metabolism (Fig. 2).

Comparing the *P*‐values of differentially expressed genes with the findings from five previous aging transcriptomics studies yielded Spearman rank correlation coefficients from 0.44 to 0.72. The overlap in the direction of the fold change of each gene ranged from 65% to 100% per gene (Table 2, Fig. S1, Supporting information). One specific gene, namely *LRRN3* (Leucine Rich Repeat Neuronal 3), was identified to be the most significantly regulated gene by all studies including the present one. *LRRN3* was consistently found to be downregulated in older and/or LLI.

**Table 1:** Overview of study participants

	Number of individuals	Age range (years)	Gender (f/m)	Country
Long‐lived individuals (LLI)	55	90–104	40/15	GER
Control individuals (CI)	73	20–55	45/28	GER
Long‐lived twins (LLT)	48 (28 DZ, 20 MZ)	83–92	32/16	DK
Control twins (CT)	48 (24 DZ, 24 MZ)	58–60	32/16	DK
Unrelated LLI	10	83–92	8/2	DK
Unrelated CI	10	58–60	4/6	DK

Country: Germany (GER), Denmark (DK).

DZ: dizygotic, MZ: monozygotic.

### eQTL and genotype–age (G×A) interaction analysis of the differentially expressed genes

**Figure 3:**
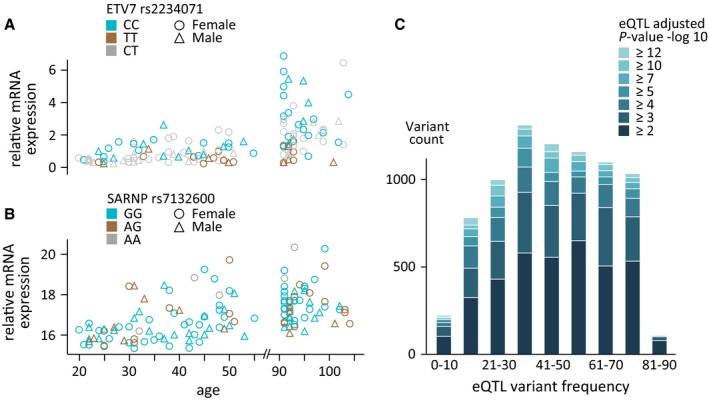
**Age‐associated transcripts and influence of genetic variation. One under genetic control (A,** ***ETV*** ***7*) and one independent of genetic control (B,** ***SARNP*** **), while** ***ETV*** ***7* shows an additional age–genotype interaction. Frequencies of** **eQTL** **variants (C) are shown color‐coded with the corresponding** ***P*** **‐value (‐log**_**10**_ ***P*).**

After quality control, 636 904 SNPs were used for the eQTL analysis in the German sample. The *cis*‐eQTL analysis identified 8757 statistically significant *cis*‐eQTLs associated with 1200 differentially expressed genes (Fig. 3, Table S3, Supporting information). A subsequent G×A analysis showed that 599 of these 1200 genes displayed an additional G×A interaction (Table S4, Supporting information). An exemplary gene exhibiting G×A interaction is shown in Fig. 3A and an example for a gene with no GxA and no significant eQTL finding is shown in Fig. 3B. The analysis of the frequency of the variants revealed that the majority (25^th^ to 50^th^ percentile) of significant eQTL signals are found in 20–50% of all individuals (Fig. 3C).

**Figure 4:**
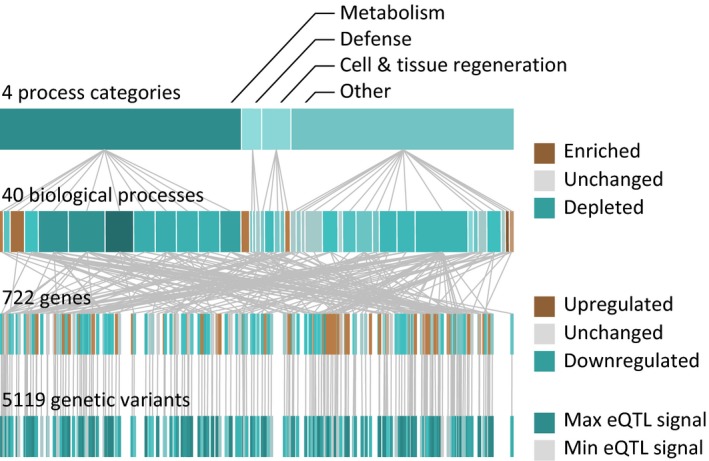
**Hierarchical organization of** **eQTL** **effects on longevity. Four different layers are represented with their hierarchical connections: 5119 variants influencing the longevity‐associated** **mRNA** **expression of 722 genes, contributing to 40 biological processes, which are grouped in four categories. The graph is based on biological processes that contain at least 150 genes significantly associated with longevity** (corresponding to Fig. 2). **For better readability, the number of displayed connections between two hierarchical levels was limited to a maximum of 200. The** ***y*** **‐axis corresponds to the hierarchical level, while the** ***x*** **‐axis represents the genomic location of genes and variants.**

Integrating the identified biological processes with the transcriptome and genotype data results in a complex hierarchical organization. To illustrate this architecture, the complexity was reduced by including only biological processes that contain 150 or more eQTL genes. We found that 5119 variants influence 722 genes (eQTLs), further contributing to 40 biological processes that can be grouped into four major biological categories, all of them affecting the longevity phenotype (Fig. 4).

Finally, comparing our eQTL findings with previously published genetic variants associated with age‐related diseases (GWAS catalog), we did not observe an enrichment of these variants in our set of identified eQTLs, keeping in mind that our experimental setup may not be suitable to capture the enormous genetic variability of this trait.

### Technical validation of selected differentially expressed genes

We replicated our results from the differential expression analysis by measuring the expression levels of 80 candidates (selected based on fold change, significance of differential expression, and eQTL *P*‐value, Table S6, Supporting information) employing RT‐qPCR in the German samples (53 LLI and 70 CI; five samples were excluded due to lack of RNA). Of the 80 genes, 74 were detectable, of which 50 were significantly differentially expressed between LLI and CI. Moreover, these 50 genes were regulated in the same direction as in the transcriptome sequencing data. A permutation analysis revealed that by chance, one would have expected 0.042 of the 80 genes to be differentially expressed; therefore, finding 50 differentially expressed genes corresponds to a high validation rate, as reflected by a low probability of detecting 50 genes by chance (Fisher's exact test *P*‐value= 7.57 × 10^−17^).

### Biological validation of differentially expressed genes and *cis*‐eQTLs

The 6075 differentially expressed genes in Germans were subjected to further validation in the Danish samples (34 older individuals [83–92 years], 34 younger individuals [58–60 years]). Of these genes, 5183 were regulated in the same direction as in the German dataset, with 1661 of those showed a Benjamini‐Hochberg corrected *P*‐value ≤ 0.05 and a FDR ≤ 5%. This concordance is significantly higher than expected by chance (Fisher's exact test, expected overlap vs. observed overlap; *P*‐value = 2.4 × 10^−13^). A gene ontology analysis of these 1661 genes found that nine of the 25 most significant biological processes were functionally linked to metabolism, which is in concordance with the findings in the German samples.

We further correlated the transcriptome and genotype data from the 68 Danish samples to validate the *cis*‐eQTLs found in the German dataset. Of the 1200 genes, which exhibited a significant association with *cis*‐variants in the German sample, 374 genes showed significant *cis* association in the Danish dataset as well. In total, we found 3332 statistically significant *cis*‐eQTLs associated with these 374 genes, of which 2017 were unique to the Danish dataset.

### Heritability estimation of gene expression

Of the 24 934 genes described in the annotation file (hg19), 15 640 were expressed in more than 95% of the blood samples of monozygotic (MZ) and dizygotic (DZ) twins from Denmark (Appendix S2, Supporting information, raw FPKM‐values for all detected genes: Supplemental raw data, Danish samples). Of these, 3029 genes are controlled by a heritable component, which ranges from approximately 30% to 99% (Table S5, Supporting information). Combining this with the above‐mentioned eQTL results (German dataset), an overlap of 334 genes was found: These genes were differentially expressed and associated with a genetic variant in *cis*, while at the same time also exhibited heritable transcriptional activity. By chance, one would have expected 182 genes to overlap. The observed 1.8‐fold enrichment was highly significant (Fisher's exact test, *P* = 1.90 × 10^−36^).

## Discussion

Understanding the nature of human longevity is a challenging task, as this heterogeneous phenotype is influenced by both genetic and environmental components. Conservative genetic approaches in longevity research have resulted so far in only very few associations that were consistently replicated in various samples (Schächter *et al*., 1994; Willcox *et al*., 2008; Flachsbart *et al*., 2009; Nebel *et al*., 2011; Deelen *et al*., 2014). The majority of genetic findings could not be verified independently (Christensen *et al*., 2006), suggesting that a large number of different variants may lead to the same phenotypic outcome and rendering it difficult to identify susceptibility factors shared by several populations. To overcome the limitation of a purely genetic study design, some investigations focused on the transcriptome of LLI that reflects both environmental and genetic influences. In the present study, this approach was expanded by integrating, for the first time in longevity research, RNA‐sequencing data from blood samples of 244 LLI and younger controls with genotype information from the same individuals, followed by a heritability analysis in twins. Employing this setup, the study's objective was to identify biological processes associated with longevity and to assess the genetic and nongenetic contribution to those.

In contrast to previous studies, a substantial higher number of genes were observed to be differentially expressed. This observation may be mostly attributed to the high dynamic range of the applied RNA‐sequencing technology (Zhao *et al*., 2014). The top candidates identified here (*LRRN3*,* P* = 5.40 × 10^−20^; *ABLIM1*,* P* = 2.50 × 10^−11^; *CD248*,* P* = 1.50 × 10^−23^, see Table S1, Supporting information) were in agreement with previously published findings (Hong *et al*., 2008; Harries *et al*., 2011; Passtoors *et al*., 2012; Van den Akker *et al*., 2014; Peters *et al*., 2015), which confirms the validity of our setup (Table 2, Fig. S1, Supporting information). The overlap between previous findings with the results presented here is illustrated in Fig. S2 (Supporting information). The top three genes, prominently identified by all previous studies, are shown with their cross‐sectional mRNA expression in Fig. S3 (Supporting information, *ABLIM1, CD248, LRRN3*). An earlier blood‐based eQTL meta‐study (Westra *et al*., 2013), despite its large sample size, is not suitable to display longevity effects, as the samples were composed of continuous age‐ranges, excluding extremely long‐lived individuals. Consequently, the previously observed effects reflect mostly aging, while the results presented here are likely to be a combination of both aging and longevity. To critically assess the power of our analysis, we conducted a permutation‐based power estimation, illustrating that the study setup selected is appropriate to deliver significant results and therefore appropriate to support our conclusions (Fig. 1B). Similarly, the successful technical validation of selected genes by quantitative real‐time PCR documents that RNA‐sequencing is a precise method for identifying differentially expressed genes as indicated by the low probability of our results being validated by chance (*P*‐value = 7.57 × 10^−17^). In the same context, the biological validation of findings from the German study population in Danes showed a high concordance (*P*‐value of this overlap not occurring by chance: 2.4 × 10^−13^).

**Table 2:** Overlap with previous studies in aging/longevity transcriptomics

	Hong	Harries	Passtoors	Akker	Peters
Study year	2008	2011	2012	2014	2015
Overlap	100%	100%	98%	65%	73%
Overlap *P*‐value	3.2 × 10^−14^	3.8 × 10^−51^	6.9 × 10^−154^	<1.0 × 10^−300^ a	<1.0 × 10^−300^ a
SRCC	0.73	0.62	0.60	0.44	0.61
Shared genes in top 10	FCGBP LRRN3^b^ NRCAM PDGFRB	ABLIM1 CCR7 CD248 FAM102A LRRN3^b^ NELL2	ABLIM1 CAMK4 CD248 LRRN3^b^ NOG	LRRN3^b^ NELL2	ABLIM1 CD248 FAM102A LRRN3^b^ NELL2

The following publications were used to generate this table: Hong (Hong *et al*., 2008), Harries (Harries *et al*., 2011), Passtoors (Passtoors *et al*., 2012), Akker (Van den Akker *et al*., 2014), and Peters (Peters *et al*., 2015). The overlap was calculated based on how many identified genes were significantly regulated and concordant in their regulation direction. Spearman rank correlation coefficient (SRCC) was calculated using all genes identified in the corresponding study. Shared genes in top 10 describes which genes were found in both studies to be among the top 10 most significantly regulated transcripts, ranked by false discovery rate (Van den Akker) or by *P*‐value (all others).

a Fisher's exact test *P*‐value could not be calculated (*P* < 1.0 × 10^−300^).

b *LRRN3* (Leucine Rich Repeat Neuronal 3) was identified as the most significantly regulated gene by all studies including the present one.

Quantitative data from whole blood are known to be influenced by altered cell compositions, yet focusing on only one selected cell population in blood might not be sufficient to create a broad picture of longevity‐associated transcriptome patterns, as it is unclear which cell type contributes dominantly to these patterns. Moreover, whole blood has been shown to reflect the age‐related molecular changes seen in other tissues quite well (Passtoors *et al*., 2008). The presented approach, that is, omitting all genes that show a high correlation with specific blood cell types (Palmer *et al*., 2006), introduces a degree of robustness, because it removes all signals which are likely to fluctuate as a result of age‐related cell composition changes.

Further analysis revealed that about 20% of the identified differentially expressed genes were influenced by *cis*‐variants (Table S3, Supporting information), while about half of those exhibited an additional genotype–age interaction: The same genotype exerts a different effect at different ages (Table S4, Supporting information, exemplified in Fig. 3A). Interestingly, we could not find an enrichment of genetic variants associated with age‐related phenotypes such as Parkinson's disease, type II diabetes, or others in our group of *cis*‐variants.

Monitoring rare or private variants in longevity is not possible with the setup presented here. Consequently, our approach can focus only on shared effects: The majority of eQTL signals occur in 20 to 50% of all individuals (Fig. 3C). This is in concordance with a previous large‐scale eQTL study by the GEUVADIS consortium, employing 462 transcriptomes with corresponding genomes, where the majority of eQTLs identified were shared as well (Lappalainen *et al*., 2013).

As each gene can potentially interact with all the detected variants (730 525 SNPs) in *trans*, we categorized all non‐*cis* interactions as nongenetic: In any given experimental setup, *trans*‐interactions are almost undetectable, despite their presence and potential relevance. We are aware that categorizing the remaining 80% as nongenetic is likely to be an overestimation due to the contribution of *trans*‐regulatory effects. Keeping this in mind, the conclusion that 80% of the genes were not under *cis*‐regulation still supports the notion that longevity is influenced to a large extent by nongenetic factors (Talens *et al*., 2012).

The genetic influence observed here was further emphasized by the subsequent heritability analysis revealing that only 19% of all genes are influenced by detectable heritability (Table S5, Supporting information). Moreover, the variation in those heritable genes was explained by heritability in a range from 30% to 99%. This highlights that most genes with a heritable component are under additional control of nongenetic factors, while the majority of genes show no heritable influence. Consequently, it appears there is no biological process that is independent of a nongenetic contribution.

Interestingly, about one‐third of the genes that were influenced by *cis*‐variants also displayed a significant heritable component in an independent set of samples, which is substantially more than expected by chance (enrichment *P*‐value *P* = 1.90 × 10^−34^). In contrast to that, several genetic variants might require additional environmental or genetic effects to display a functional impact on mRNA expression, explaining why a large number of genes that were influenced by *cis*‐variants did not show a heritable component. Finally, our observations on the heritability on the molecular level are in concordance with previous findings on the phenotype level of familial aggregation of longevity as reviewed in Murabito *et al*., 2012.

As a primary result of the subsequent gene ontology analysis, we found 107 biological processes associated with longevity, most of which were assigned to one of the three categories: metabolism, tissue and cell regeneration as well as immune system and defense. Within this, the most prominent finding is the significant depletion of processes functionally linked with metabolism (Fig. 2), which dominates by number of genes per process (median: 1103) as well as by significance (min. *P* = 3.5 × 10^−24^). Keeping the limitations of pathway‐analysis approaches in mind, the unusually large number of genes associated with the individual metabolic process (1085–1555) further increases our confidence in this finding. Here, the large number of metabolism‐associated processes identified might indicate a key role of this process category in longevity. Modulations in cell and tissue regeneration and in defense processes were significant as well, yet less dominant as the number of genes contributing to these observations was substantially lower. The depletion of metabolic processes resulted mostly from a downregulation of metabolism‐associated genes in both the German and Danish LLI. The downregulation may point to an existing reduced (resting) metabolic rate that in turn has been linked with increased lifespan in humans (Ruggiero *et al*., 2008; Rozing *et al*., 2010). Unfortunately, no metabolic rate data were available for our study participants.

Our results support the hypothesis that a subgroup of individuals, exhibiting patterns of reduced metabolism (whether due to genetic and/or environmental factors), is more likely to reach old age in a healthy state. An interesting question that arises in this context is if these patterns in LLI are a consistent feature throughout their lives or are they restricted only to advanced age? The approach applied here using cross‐sectional data does not allow us to address this issue. If the former was the case, the younger individuals that cluster with the LLI based on downregulation of metabolism‐associated genes (highlighted in Fig. 1A) might represent good candidates for people living up to their nineties and beyond.

It is not unlikely that the downregulation of metabolism‐associated genes illustrates the lifestyle of the LLI when compared to younger controls, attributed to moderate food intake and reduced physical activity (Von Wurmb‐Schwark *et al*., 2010). In this context, it is important to mention the hypothesis of longevity being linked to caloric restriction, which is supported by several prominent observations: For example in Okinawans, caloric restriction and traditional functional food were suggested to play a role in extended lifespan (Willcox & Willcox, 2014). Similarly, caloric restriction has been proposed to have beneficial effects for age‐related outcomes in the CALERIE cohort (Ravussin *et al*., 2015). While our findings seem to further support this hypothesis, our data do not allow creating a direct link to caloric restriction, as we have demonstrated a depletion of metabolic processes without measuring metabolic rates or caloric intake.

Considering that the majority (~80%) of the observed depletion of metabolic processes is independent of genetics, one could assume that this is indeed the result of longevity. In contrast to this, the genetic contribution (~20%) to this depletion allows a second implication: Individuals with a given genetic setup will have an altered metabolic pattern, resulting in a higher probability of reaching old age. Therefore, our eQTL findings support the concept that longevity is not only a consequence of environmental factors, but also driven by genetics. Moreover, the complexity of the genetic contribution is further increased by the fact that some variants have different impacts at different ages, as demonstrated by our findings on genotype–age interaction (Fig. 3).

It is important to note that the observed depletion of metabolic processes has limited predictive value for other samples: The cross‐sectional study design employed here may overestimate this depletion by reflecting differences between birth cohorts, yet the correlation between altered expression and increasing chronological age (Fig. 3A, Fig. S4, Supporting information) supports a noncohort‐specific effect. This is facilitated by the large age windows covered by our cohorts (see also Fig. S4, Supporting information); our study setup neither allows nor aims at monitoring aging effects but instead is designed to identify characteristics of long‐lived individuals. In contrast to that, an alternative longitudinal setup suffers similar limitations as findings in longitudinal studies potentially have validity only for the birth cohort investigated. Keeping the limitations of the cross‐sectional study design in mind, the integration of stable genomic data with dynamic transcriptome data from different age windows allows us to illustrate the functional consequences of genetic variations. As the SNPs observed in the eQTLs cannot be the result of longevity, this approach offers the unique opportunity to identify potentially causative factors contributing to the phenotype.

Finally, our results might also partially explain why replication of genetic longevity associations is a challenging task. We observed a hierarchical structure, where a large number of heterogeneous heritable and nonheritable, genetic and nongenetic components potentially lead to altered pathway patterns, which finally may result in longevity. The heterogeneity of factors contributing to the same phenotype, as illustrated in Fig. 4, might explain why some genetic associations appear to be valid only in a specific population. Moreover, the independent cohort from Denmark we employed to biologically validate our findings in German individuals displayed the same modulation in metabolic pathways, yet the genes and variants of origin did only partially overlap between Danes and Germans. This provides further support for the concept of a hierarchical structure, where different genetic variations lead to the same phenotype.

Taken together, our systems biology‐based analysis on unique longevity samples illustrates, for the first time, how human longevity is associated with a depletion of metabolic pathways in a genotype‐dependent and independent manner. At the same time, our eQTL findings indicate that those processes are, to some extent, also a driving force of longevity rather than only a consequence of old age.

## Experimental procedures

### Study populations

This study included a total of 244 whole blood samples from LLI and control individuals (CI) (Table 1). The samples of the German subjects were collected with the support of the biobank PopGen, as previously described (Nebel *et al*., 2005). The Danish individuals were recruited from two population‐based and nationwide twin surveys conducted at the Danish Twin Registry: the Longitudinal Study of Middle‐aged Danish Twins and the Longitudinal Study of Aging Danish Twins (Skytthe *et al*., 2013) (Table 1). All participants signed a written informed consent. Approval for the study was received from the Ethics Committees of Kiel University and the Regional Scientific Ethical Committees for Southern Denmark.

### Sample processing

Total RNA was extracted for all 244 individuals from frozen blood samples using the PAXgene Blood miRNA kit (Qiagen) according to the manufacturer's protocol. Paired‐end libraries were prepared with the Illumina TruSeq RNA Sample Preparation Kit, multiplexed with four samples per lane and sequenced on an Illumina HiSeq 2000.

For the German study population, DNA was extracted from EDTA whole blood using the Invisorb Blood Giga Kit following the manufacturer's instructions. DNA samples from all German individuals (55 LLI and 73 CI) were subjected to genotyping employing the HumanOmniExpress BeadChip (Illumina) that monitors 730 525 SNPs. For the Danish population, DNA was extracted by a salting‐out procedure either performed manually or with the AutoPure LS instrument (Qiagen). One individual from each of the 22 MZ pairs (LLT = 10 pairs and CT = 12 pairs) as well as 52 DZ twin individuals (LLT = 14 pairs and CT = 12 pairs) and 20 unrelated individuals (10 LLI and 10 CI) were selected for genotyping.

### Data analysis

The sequencing reads that failed the Illumina chastity filter (chastity threshold = 0.6) were removed with the Illumina CASAVA‐1.8 FASTQ filter v0.1. Subsequent alignment to the UCSC *Homo sapiens* reference genome (build hg19) was performed with TopHat v2.0.4 (Trapnell *et al*., 2009). The aligned reads were assembled into transcripts and gene‐level abundance in terms of fragments per kilobase of exon per million fragments mapped (FPKM) was estimated by Cufflinks v2.0.2 (Trapnell *et al*., 2010). After quantile sample‐to‐sample normalization, the FPKM estimates from the German samples were used to identify the differentially expressed genes. Only genes with a log2 FPKM > 0 in more than 5% of the samples were subjected to further analysis, while differential expression was determined using a nonparametric Wilcoxon rank sum test. Genes with a corrected *P*‐value ≤ 0.001 (employing Benjamini and Hochberg's correction) and a false discovery rate (FDR)  ≤ 0.1% based on a Westfall and Young permutation of the fold changes were considered to be significantly differentially expressed. Differentially expressed genes that highly correlated (*r* ≥ 0.8) with blood cell counts (Palmer *et al*., 2006) were omitted from further analysis.

Gene expression data from the Danish twins were used to estimate the heritability of transcriptional activity. Only genes that were expressed (i.e., log2 FPKM > 0) in more than 95% of the samples were subjected to heritability estimation that assessed additive genetic effects. An ACE model (A: additive genetics, C: common environment, E: unique environment) was fitted using biometric modeling (R package *mets* v0.1‐13) and was allowed to compete with the parsimonious nested models, namely AE, CE, and E. The best fitting model was chosen based on the Akaike information criterion (AIC) (Akaike, 1974) for non‐nested models and the likelihood ratio test for nested models. The final heritability estimate was obtained from the best fitting model.

Genes that were differentially expressed between LLI and CI (German dataset) were subjected to *cis*‐eQTL analysis. Only variants located within a 1‐Mb up‐ and downstream of the starting and end points of the gene were included in the analysis. SNPs with minor allele frequencies below 1%, call rate below 95%, and Hardy–Weinberg equilibrium testing *P*‐value ≤ 0.0001 were filtered out. The eQTL analysis for the German samples was performed with PLINK v1.07 (Purcell *et al*., 2007). The obtained *cis*‐eQTLs were adjusted using a Benjamini and Hochberg correction for multiple testing at an α level of 5%. Furthermore, the SNPs that exhibited significant association with gene expression (*cis*‐eQTLs) were compared with genetic variants from GWAS catalog (https://www.ebi.ac.uk/gwas/) to examine whether there is an enrichment of variants associated with age‐related diseases in our identified eQTLs. To assess the frequency of variances per gene, all significant eQTL findings were binned according to their occurrence in the German study population.

To assess the genotype–age interaction (G×A), only differentially expressed genes with at least one significant *cis*‐eQTL were considered. The G×A interaction for each gene and the most significant cis‐eQTL were investigated by employing a linear mixed model as described previously (Glass *et al*., 2013).

The overlap with previous studies (Hong *et al*., 2008; Harries *et al*., 2011) was assessed by transforming the observed *P*‐values to −log(P,100), multiplied with the sign of the fold change, to properly reflect the direction of regulation. For one study (Harries *et al*., 2011), only the false discovery rate data were available which was used equivalently. Subsequently, the transformed *P*‐values of the genes that were found in both our study and the previous one were subjected to a correlation analysis using the Spearman rank correlation. The expected overlap between studies was based on the number of identified genes in each study, assuming a genomewide approach. The overlap *P*‐value was calculated employing a Fisher's exact test comparing observed vs. expected overlap.

### Functional analysis: gene ontology

Gene ontology analysis was performed by associating genes of interest to gene ontology terms (retrieved from http://www.geneontology.org), followed by a two‐sided Fisher's exact test to determine significance of enrichment or depletion. Finally, resulting *P*‐values were adjusted employing a Benjamini and Hochberg's correction for multiple testing.

### Technical validation of differential gene expression by real‐time PCR

Eighty candidate genes were selected based on the ranks of their fold changes, *P*‐values, and regulation of their expression by *cis*‐eQTLs and were subjected to validation in the German samples. Real‐time PCR (TaqMan) was performed according to the manufacturer's instructions (Applied Biosystems) on a 7900HT real‐time PCR system. Gene expression levels were quantified relative to the median of three genes (*RER1*,* E2F4*,* BFAR*) that showed minimum variation in the expression levels across the samples. Differences between LLI and CI were determined with the Wilcoxon rank sum test and the *P*‐values were corrected using Benjamini and Hochberg's method.

### Biological validation of differential gene expression and *cis*‐eQTLs using Danish samples

In total, 68 individuals were included in the validation set (10 unrelated LLI, 10 unrelated controls, and 48 individuals selected from 48 twin pairs: 22 MZ and 26 DZ – Table 1). The RNA‐sequencing‐based transcriptome data from these 68 samples were analyzed as described above to identify and validate the differentially expressed genes detected in the German dataset. As the validation dataset was smaller than the German sample, the genes with a corrected *P*‐value ≤ 0.05 and a FDR ≤ 5% were considered to be significantly differentially expressed. The genotype and gene expression data were analyzed using PLINK.

## Funding

The Danish Twin Registry is supported by grants from The National Program for Research Infrastructure 2007 (09‐063256) from the Danish Agency for Science Technology and Innovation, the Velux Foundation, and the US National Institute of Health (P01 AG08761). This study was funded by INTERREG 4A Syddanmark‐Schleswig‐K.E.R.N., the Excellence Cluster ‘Inflammation at Interfaces’ (DFG), the PopGen 2.0 network (BMBF, 01EY1103), and by the German National Genome Research Network (NGFN, BMBF).

## Author contributions

RH participated in designing the study, supervising the analysis, and writing the manuscript. GV carried out the analysis and contributed to writing the manuscript. QT participated in the analysis and supervised the analysis. FF participated in designing and coordinating the study. PR participated in the sequencing analysis, coordinating the study, and drafting the manuscript. WL contributed to the study design and the analysis. SS, KC, LC, and AN participated in study design, coordination of the study, and drafting and finalizing the manuscript.

## Conflict of interest

All authors read and approved the final manuscript and declare no conflict of interest.

## Supporting information


**Fig. S1** Comparison of findings to previous transcriptomic studies.

**Fig. S2** Unique and overlapping features compared to previous studies.

**Fig. S3** Cross sectional mRNA regulation of three selected genes.
**Fig. S4** mRNA expression of genes correlating with age.
**Table S1** The top 25 up‐ and downregulated genes in LLI.
**Table S2** All the significantly represented biological processes with respect to the genes upregulated and downregulated in LLI (in the German samples).
**Table S3** The top 50 *cis*‐eQTLs associated with differentially expressed genes.
**Table S4** The top 50 G×A interaction effects exhibited by differentially expressed genes.
**Table S5** The top 50 genes with heritable transcriptional activity; the best fit model for these genes was AE (A=additive genetic effect, E=unique environmental effect).
**Table S6** List of 80 candidate genes and the TaqMan assays used for validation.

**Appendix S1** This file contains FPKM (fragments per kilobase of exon per million fragments mapped) values for all German samples.

**Appendix S2** This file contains FPKM (fragments per kilobase of exon per million fragments mapped) values for all Danish samples.
