# Supplementary figures and images for: Genetic interplay between human longevity and metabolic pathways — a large‐scale eQTL study
Source: Aging Cell. 2017 Apr 19;16(4):716–25. doi: 10.1111/acel.12598 (PMC5506416; doi:10.1111/acel.12598)

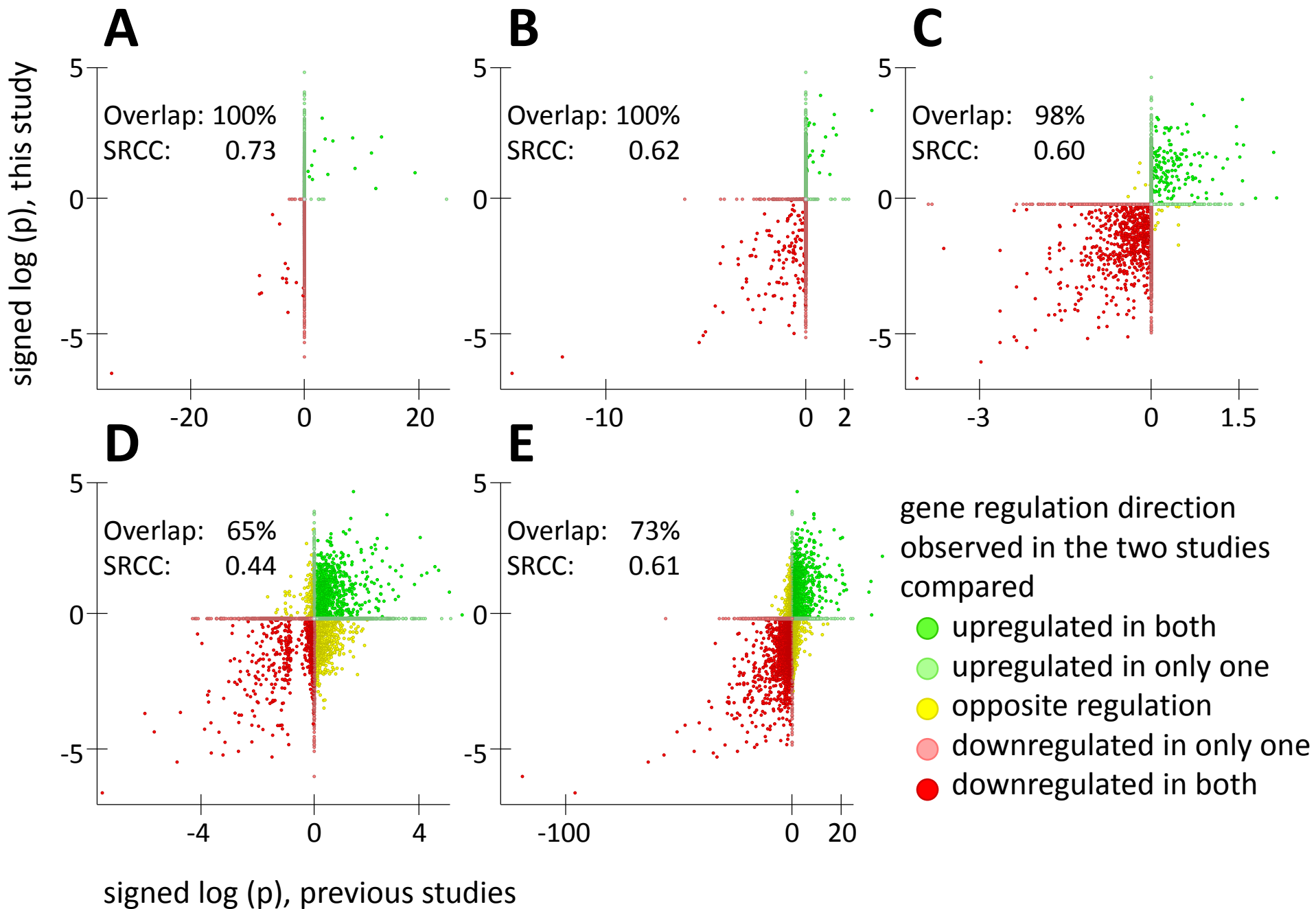

Supplement: Supplementary file 1 — Fig. S1 Comparison of findings to previous transcriptomic studies. [file ACEL-16-716-s001.pdf]

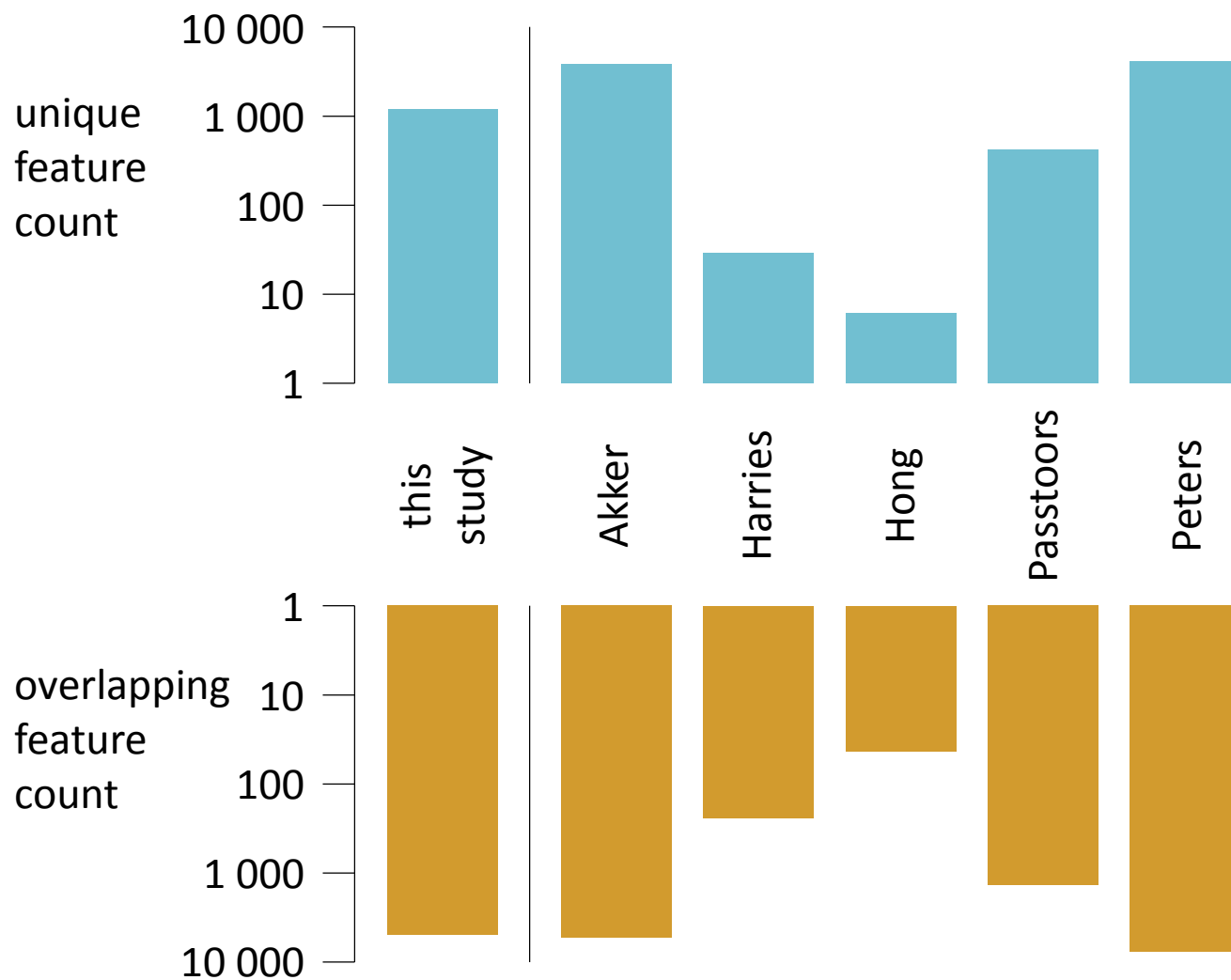

Supplement: Supplementary file 2 — Fig. S2 Unique and overlapping features compared to previous studies. [file ACEL-16-716-s002.pdf]
