# Supplementary material for: Genetic interplay between human longevity and metabolic pathways — a large‐scale eQTL study
Source: Aging Cell. 2017 Apr 19;16(4):716–25. doi: 10.1111/acel.12598 (PMC5506416; doi:10.1111/acel.12598)
Supplement: Supplementary file 3 — Fig. S3 Cross sectional mRNA regulation of three selected genes. Fig. S4 mRNA expression of genes correlating with age. Table S1 The top 25 up‐ and downregulated genes in LLI. Table S2 All the significantly represented biological processes with respect to the genes upregulated and downregulated in LLI (in the German samples). Table S3 The top 50 cis‐eQTLs associated with differentially expressed genes. Table S4 The top 50 G×A interaction effects exhibited by differentially expressed genes. Table S5 The top 50 genes with heritable transcriptional activity; the best fit model for these genes was AE (A=additive genetic effect, E=unique environmental effect). Table S6 List of 80 candidate genes and the TaqMan assays used for validation. [file ACEL-16-716-s003.docx]

# SUPPLEMENTAL MATERIAL

# Supplementary Figure 1: Comparison to previous transcriptomic studies

Differentially expressed genes are displayed based on their signed log (P), where a negative log(P) corresponds to the significance of downregulated genes, while a positive log(P) corresponds to upregulated genes. The overlap is based on the degree of concordance in regulation direction for overlapping findings. Similarly, the spearman rank correlation coefficient (SRCC) was calculated only for genes identified in both studies. Genes are coloured according to their concordance in regulation direction with previous studies A: Hong et al, 2008; B: Harries et al, 2011; C: Passtoors et al, 2012; D: Van den Akker et al, 2014 and E: Peters et al, 2015.

# Supplementary Figure 2: Unique and overlapping features compared to previous studies.

The total count of genes unique (upper part) and overlapping (lower part) features per study is displayed on a logarithmic scale. A differentially expressed gene is categorized in unique in a study, if none of the other five studies detected this feature. Similarly, a differentially expressed gene is considered as overlapping, if at least one of the other five studies observed this gene as differentially expressed as well. Studies are labelled by their first author: Hong et al, 2008; Harries et al, 2011; Passtoors et al, 2012; Van den Akker et al, 2014 and Peters et al, 2015.

# Supplementary Figure 3: Cross sectional mRNA regulation of three selected genes

identified in concordance with previous studies (Hong et al, 2008; Harries et al, 2011; Passtoors et al, 2012; Van den Akker et al, 2014 and Peters et al, 2015). A: Actin Binding LIM Protein 1 (ABLIM1); B: CD248 Molecule (CD248); C: Leucine Rich Repeat Neuronal 3 (LRRN3).

# Supplementary Figure 4: mRNA expression of genes correlating with age.

Relative mRNA expression (quantile scaled) versus chronological age of the top five genes with negative correlation (A-E) and top five genes with positive correlation (F-J), strongest correlation first. The gap in the x-axis results from the two distinct age groups compared. The trend line represents the linear curve-fit of the data presented, while the gap was ignored for better visualization. Spearman rank correlation coefficients are displayed for each gene separately.

# Table S1: The top 25 up- and downregulated genes in LLI

| Gene symbol | Gene name | | Adjusted_pvalue | Fold change |
| --- | --- | --- | --- | --- |
| Upregulated genes | | |  |  |
| DNAJC1 | | DnaJ (Hsp40) homolog, subfamily C, member 1 | 3.12E-13 | 1.593 |
| LOC100271722 | | encoding hypothetical LOC100271722 | 9.38E-12 | 1.817 |
| PHLDA3 | | pleckstrin homology-like domain, family A, member 3 | 1.36E-11 | 3.143 |
| ANKRD22 | | ankyrin repeat domain 22 | 1.40E-11 | 2.913 |
| ODF3B | | outer dense fiber of sperm tails 3B | 1.59E-11 | 2.102 |
| FCGR1A | | Fc fragment of IgG, high affinity Ia, receptor (CD64) | 1.67E-11 | 3.207 |
| MS4A4A | | membrane-spanning 4-domains, subfamily A, member 4A | 3.08E-11 | 2.491 |
| SAP30 | | Sin3A-associated protein, 30kDa | 6.73E-11 | 1.740 |
| POLB | | polymerase (DNA directed), beta | 2.03E-10 | 1.707 |
| PSMB8 | | proteasome (prosome, macropain) subunit, beta type, 8 | 2.27E-10 | 1.237 |
| VAMP5 | | vesicle-associated membrane protein 5 | 2.27E-10 | 2.163 |
| PAQR4 | | progestin and adipoQ receptor family member IV | 2.81E-10 | 1.717 |
| ZBP1 | | Z-DNA binding protein 1 | 3.12E-10 | 1.480 |
| FCGR1C | | Fc fragment of IgG, high affinity Ic, receptor (CD64),  pseudogene | 3.66E-10 | 3.575 |
| TRIP4 | | thyroid hormone receptor interactor 4 | 4.45E-10 | 1.441 |
| SLC27A3 | | solute carrier family 27 (fatty acid transporter), member 3 | 4.50E-10 | 1.304 |
| HOXB7 | | homeobox B7 | 4.70E-10 | 3.503 |
| DES | | Desmin | 4.71E-10 | 2.899 |
| PDGFRB | | platelet-derived growth factor receptor, beta polypeptide | 8.29E-10 | 2.629 |
| MANEAL | | mannosidase, endo-alpha-like | 8.60E-10 | 2.473 |
| RIPK3 | | receptor-interacting serine-threonine kinase 3 | 8.93E-10 | 1.448 |
| BATF2 | | basic leucine zipper transcription factor, ATF-like 2 | 9.56E-10 | 2.925 |
| FAM20A | | family with sequence similarity 20, member A | 9.66E-10 | 2.419 |
| LGALS1 | | lectin, galactoside-binding, soluble, 1 | 1.01E-09 | 1.830 |
| SQRDL | | sulfide quinone reductase-like (yeast) | 1.22E-09 | 1.261 |
| Downregulated genes | | | |  |
| LRRN3 | leucine rich repeat neuronal 3 | | 9.03E-17 | -9.112 |
| CD248 | CD248 molecule, endosialin | | 1.57E-15 | -5.469 |
| NELL2 | NEL-like 2 (chicken) | | 1.82E-14 | -2.332 |
| NOG | Noggin | | 4.46E-14 | -3.949 |
| ABLIM1 | actin binding LIM protein 1 | | 6.13E-14 | -2.320 |
| NT5E | 5'-nucleotidase, ecto (CD73) | | 8.94E-14 | -2.841 |
| CAMK4 | calcium/calmodulin-dependent protein kinase IV | | 1.19E-13 | -2.060 |
| FAM102A | family with sequence similarity 102, member A | | 1.19E-13 | -2.000 |
| RCAN3 | RCAN family member 3 | | 2.30E-13 | -2.099 |
| RASGRF2 | Ras protein-specific guanine nucleotide-releasing factor 2 | | 3.42E-13 | -2.431 |
| SERPINE2 | serpin peptidase inhibitor, clade E (nexin, plasminogen activator  inhibitor type 1), member 2 | | 3.42E-13 | -2.790 |
| NPAS2 | neuronal PAS domain protein 2 | | 3.70E-13 | -4.513 |
| SPTBN1 | spectrin, beta, non-erythrocytic 1 | | 3.70E-13 | -2.269 |
| N4BP3 | NEDD4 binding protein 3 | | 5.59E-13 | -2.631 |
| SFRP5 | secreted frizzled-related protein 5 | | 5.59E-13 | -12.998 |
| KLHL3 | kelch-like family member 3 | | 5.85E-13 | -1.788 |
| CHMP7 | charged multivesicular body protein 7 | | 7.04E-13 | -1.591 |
| GPRASP1 | G protein-coupled receptor associated sorting protein 1 | | 7.26E-13 | -2.340 |
| CACHD1 | cache domain containing 1 | | 9.12E-13 | -5.505 |
| NIPAL3 | NIPA-like domain containing 3 | | 1.48E-12 | -1.501 |
| ZNF549 | zinc finger protein 549 | | 1.82E-12 | -1.490 |
| AQP3 | aquaporin 3 (Gill blood group) | | 2.12E-12 | -2.174 |
| EDAR | ectodysplasin A receptor | | 3.08E-12 | -3.476 |
| ZFYVE9 | zinc finger, FYVE domain containing 9 | | 3.08E-12 | -1.733 |
| CCR7 | chemokine (C-C motif) receptor 7 | | 3.40E-12 | -2.111 |

Adjusted_pvalue=p-value corrected for multiple testing

# Table S2: All the significantly represented biological processes with respect to the genes upregulated and downregulated in LLI (in the German samples).

| Biological processes | more/fewer genes than expected by chance | Adjuted_pvalue |
| --- | --- | --- |
| With respect to the upregulated genes | | |
| oxidation-reduction process | + | 6.58584E-11 |
| regulation of developmental process | - | 7.07123E-07 |
| generation of precursor metabolites and energy | + | 3.10797E-06 |
| regulation of cellular process | - | 3.10797E-06 |
| small molecule metabolic process | + | 1.5728E-05 |
| embryonic morphogenesis | - | 1.5728E-05 |
| regulation of signaling | - | 4.08626E-05 |
| pattern specification process | - | 7.01662E-05 |
| regulation of metabolic process | - | 8.71359E-05 |
| organ morphogenesis | - | 0.000220242 |
| cell-cell adhesion | - | 0.000233777 |
| cell adhesion | - | 0.000235619 |
| cell migration | - | 0.000235619 |
| cellular response to stress | + | 0.001547862 |
| regulation of body fluid levels | - | 0.001547862 |
| tube morphogenesis | - | 0.001547862 |
| cellular developmental process | - | 0.001559711 |
| tissue morphogenesis | - | 0.001559711 |
| antigen processing and presentation of exogenous antigen | + | 0.001953199 |
| mitochondrial transport | + | 0.002082563 |
| Coagulation | - | 0.003328394 |
| cell cycle | + | 0.006790666 |
| antigen processing and presentation of peptide antigen | + | 0.006790666 |
| system development | - | 0.006790666 |
| cell differentiation | - | 0.009982644 |
| morphogenesis of a branching structure | - | 0.010505961 |
| Regionalization | - | 0.011770173 |
| glutathione derivative metabolic process | + | 0.011770173 |
| cellular component movement | - | 0.011770173 |
| response to other organism | + | 0.013280966 |
| hydrogen transport | + | 0.013280966 |
| organ development | - | 0.013280966 |
| synapse organization | - | 0.01793919 |
| single-organism catabolic process | + | 0.018980645 |
| hematopoietic or lymphoid organ development | - | 0.025171996 |
| defense response to virus | + | 0.025171996 |
| regulation of response to stimulus | - | 0.027368965 |
| signal transduction | - | 0.030708144 |
| cellular response to endogenous stimulus | - | 0.036340253 |
| response to virus | + | 0.039529868 |
| tube development | - | 0.04358095 |
| viral entry into host cell via membrane fusion with the plasma membrane | + | 0.04358095 |
| cellular catabolic process | + | 0.04358095 |
| regulation of multicellular organismal process | - | 0.049479985 |
| With respect to the downregulated genes | | |
| cellular macromolecule metabolic process | + | 1.68181E-73 |
| regulation of metabolic process | + | 7.38584E-56 |
| macromolecule metabolic process | + | 2.0363E-55 |
| nucleobase-containing compound metabolic process | + | 5.34186E-49 |
| cellular aromatic compound metabolic process | + | 7.022E-44 |
| heterocycle metabolic process | + | 1.30312E-43 |
| organic cyclic compound metabolic process | + | 8.25218E-38 |
| cellular nitrogen compound metabolic process | + | 2.4924E-37 |
| regulation of cellular process | + | 5.83663E-33 |
| cellular biosynthetic process | + | 1.58186E-27 |
| organic substance biosynthetic process | + | 2.62531E-26 |
| organelle organization | + | 4.74181E-19 |
| negative regulation of biological process | + | 2.72502E-09 |
| protein metabolic process | + | 1.6398E-08 |
| positive regulation of biological process | + | 5.03564E-08 |
| RNA transport | + | 7.07176E-08 |
| cell-cell signaling | - | 1.14189E-06 |
| modification of morphology or physiology of other organism involved in symbiotic interaction | + | 1.99594E-06 |
| cell communication | - | 3.32407E-06 |
| modification of morphology or physiology of other organism | + | 8.34453E-06 |
| immune response-activating signal transduction | + | 1.71139E-05 |
| interaction with host | + | 2.69314E-05 |
| embryo development | + | 3.83194E-05 |
| intracellular transport | + | 4.71315E-05 |
| cellular response to endogenous stimulus | + | 5.03087E-05 |
| phosphorus metabolic process | + | 7.199E-05 |
| cell cycle | + | 0.000207797 |
| macromolecule methylation | + | 0.000342191 |
| regulation of response to stimulus | + | 0.000355204 |
| protein transport | + | 0.000380449 |
| ion transport | - | 0.000380449 |
| system process | - | 0.000423557 |
| single-organism catabolic process | - | 0.000455124 |
| organic acid metabolic process | - | 0.000455124 |
| regulation of signaling | + | 0.000515664 |
| cell cycle process | + | 0.000547866 |
| cellular localization | + | 0.00060626 |
| cellular response to stress | + | 0.000641649 |
| cellular response to chemical stimulus | + | 0.000681059 |
| oxidation-reduction process | - | 0.000832884 |
| Golgi vesicle transport | + | 0.000958635 |
| regulation of cellular component organization | + | 0.001028958 |
| gene silencing | + | 0.001212995 |
| viral process | + | 0.001214434 |
| symbiosis, encompassing mutualism through parasitism | + | 0.001214434 |
| generation of precursor metabolites and energy | - | 0.002098202 |
| regulation of cellular component biogenesis | + | 0.002415636 |
| organonitrogen compound metabolic process | - | 0.003771562 |
| single-organism biosynthetic process | - | 0.004152248 |
| establishment or maintenance of cell polarity | + | 0.004802956 |
| hematopoietic or lymphoid organ development | + | 0.005683962 |
| regulation of protein stability | + | 0.006389161 |
| maintenance of location in cell | + | 0.006389161 |
| regulation of RNA stability | + | 0.007549147 |
| stem cell maintenance | + | 0.008089919 |
| cellular response to abiotic stimulus | + | 0.008513448 |
| digestion | - | 0.008858864 |
| cellular macromolecule localization | + | 0.00887381 |
| xenobiotic metabolic process | - | 0.011159448 |
| positive regulation of molecular function | + | 0.011159448 |
| small molecule metabolic process | - | 0.012258654 |
| regulation of blood pressure | - | 0.013099716 |
| protein localization | + | 0.013474158 |
| nuclear import | + | 0.013741009 |
| immune response-regulating cell surface receptor signaling pathway involved in phagocytosis | + | 0.013741009 |
| regulation of membrane potential | - | 0.013741009 |
| response to fibroblast growth factor stimulus | + | 0.015266984 |
| activation of innate immune response | + | 0.015346946 |
| extracellular structure organization | - | 0.018782087 |
| cell death | + | 0.031538537 |
| regulation of immune system process | + | 0.036331984 |
| cellular response to stimulus | + | 0.036331984 |
| endosomal transport | + | 0.038372379 |
| cerebral cortex development | + | 0.042836664 |
| regulation of localization | + | 0.045866007 |

Adjusted_pvalue=p-value corrected for multiple testing; + means more genes than expected by chance associated with a given process and – means fewer genes than expected by change associated with a given process.

# Table S3: The top 50 *cis*-eQTLs associated with differentially expressed genes

| rs number | SNP_position | Gene symbol | BETA | STAT | Adjusted_pvalue |
| --- | --- | --- | --- | --- | --- |
| rs11062557 | 3301706 | LOC100652846 | -6.803 | -28.16 | 1.01E-52 |
| rs3957144 | 71740691 | LRRC20 | -7.566 | -28.16 | 1.32E-52 |
| rs13390788 | 235624835 | MSL3P1 | -6.757 | -25.6 | 2.80E-48 |
| rs3100872 | 50365160 | C14orf182 | -6.524 | -24.91 | 1.63E-47 |
| rs3126184 | 50372535 | C14orf182 | -6.517 | -24.87 | 1.63E-47 |
| rs2571174 | 44934489 | LYPD3 | -7.107 | -24.97 | 1.67E-47 |
| rs3100908 | 50359489 | C14orf182 | -6.516 | -25.09 | 1.84E-47 |
| rs3100877 | 50373276 | C14orf182 | -6.516 | -24.78 | 1.84E-47 |
| rs11849760 | 50392720 | C14orf182 | -6.521 | -24.85 | 1.84E-47 |
| rs10462068 | 44186271 | CCL28 | -7.412 | -20.6 | 5.81E-39 |
| rs310007 | 81473711 | SDR42E1 | -6.238 | -20.38 | 2.42E-38 |
| rs920980 | 896983 | C5orf55 | -6.973 | -19.72 | 3.98E-38 |
| rs14389 | 917878 | C5orf55 | -6.959 | -19.76 | 3.98E-38 |
| rs7728035 | 944298 | C5orf55 | -6.976 | -19.74 | 3.98E-38 |
| rs10058912 | 948117 | C5orf55 | -6.976 | -19.75 | 3.98E-38 |
| rs16867926 | 959224 | C5orf55 | -6.973 | -19.72 | 3.98E-38 |
| rs8007947 | 90299541 | LOC400236 | -7.658 | -19.71 | 2.82E-37 |
| rs3100872 | 50365160 | L2HGDH | -5.908 | -19.3 | 8.97E-37 |
| rs3126184 | 50372535 | L2HGDH | -5.907 | -19.31 | 8.97E-37 |
| rs11849760 | 50392720 | L2HGDH | -5.921 | -19.39 | 8.97E-37 |
| rs3100877 | 50373276 | L2HGDH | -5.908 | -19.24 | 1.00E-36 |
| rs3100908 | 50359489 | L2HGDH | -5.911 | -19.26 | 1.39E-36 |
| rs2247867 | 112131164 | LOC100129269 | -6.756 | -18.76 | 4.05E-35 |
| rs7782183 | 23730983 | IGF2BP3 | -6.324 | -18.12 | 2.12E-34 |
| rs950934 | 23750270 | IGF2BP3 | -6.326 | -18.17 | 2.12E-34 |
| rs6949764 | 23852833 | IGF2BP3 | -6.322 | -18.11 | 2.12E-34 |
| rs6950415 | 23941098 | IGF2BP3 | -6.329 | -18.13 | 2.12E-34 |
| rs6689899 | 2.04E+08 | LRRN2 | -7.422 | -17.94 | 2.61E-33 |
| rs285671 | 77129325 | LMO7 | -6.51 | -17.28 | 5.83E-32 |
| rs12040661 | 32209650 | BAI2 | -6.75 | -16.79 | 1.30E-31 |
| rs12035217 | 32212514 | BAI2 | -6.75 | -16.79 | 1.30E-31 |
| rs12037019 | 32213655 | BAI2 | -6.75 | -16.79 | 1.30E-31 |
| rs12037400 | 32215178 | BAI2 | -6.742 | -16.72 | 2.85E-31 |
| rs315441 | 29977110 | ATAD5 | -5.917 | -16.33 | 3.56E-30 |
| rs7507526 | 8553322 | PEX11G | -7.06 | -16.4 | 4.68E-30 |
| rs3100908 | 50359489 | SAV1 | -3.397 | -15.37 | 4.19E-28 |
| rs3100872 | 50365160 | SAV1 | -3.389 | -15.4 | 4.19E-28 |
| rs3126184 | 50372535 | SAV1 | -3.386 | -15.39 | 4.19E-28 |
| rs3100877 | 50373276 | SAV1 | -3.386 | -15.33 | 4.19E-28 |
| rs11849760 | 50392720 | SAV1 | -3.387 | -15.3 | 4.19E-28 |
| rs418891 | 43693538 | LRRC37A4 | -1.723 | -14.7 | 1.34E-27 |
| rs17334797 | 43825912 | LRRC37A4 | -1.725 | -14.68 | 1.34E-27 |
| rs17426064 | 43828698 | LRRC37A4 | -1.725 | -14.68 | 1.34E-27 |
| rs17650901 | 44039691 | LRRC37A4 | -1.725 | -14.68 | 1.34E-27 |
| rs17651549 | 44061278 | LRRC37A4 | -1.725 | -14.68 | 1.34E-27 |
| rs1052551 | 44068924 | LRRC37A4 | -1.725 | -14.68 | 1.34E-27 |
| rs1052553 | 44073889 | LRRC37A4 | -1.725 | -14.68 | 1.34E-27 |
| rs17652121 | 44073973 | LRRC37A4 | -1.725 | -14.68 | 1.34E-27 |
| rs1052587 | 44102604 | LRRC37A4 | -1.723 | -14.7 | 1.34E-27 |
| rs7350928 | 44108100 | LRRC37A4 | -1.725 | -14.68 | 1.34E-27 |

BETA = regression coefficient; STAT = coefficient t-statistic; Adjusted_pvalue = p-value corrected for multiple testing.

# Table S4: The top 50 G×A interaction effects exhibited by differentially expressed genes

| Gene symbol | rs number | SNP_position | AIC.rd | AIC.full | p-value | Adjusted_pvalue |
| --- | --- | --- | --- | --- | --- | --- |
| LOC400236 | rs8007947 | 90299541 | 460.2554 | 403.0711 | 1.44E-14 | 4.29E-11 |
| BCO2 | rs4252588 | 111784229 | 504.9374 | 468.0975 | 4.60E-10 | 6.87E-07 |
| C16orf52 | rs11074499 | 21687108 | 463.5223 | 432.2775 | 8.13E-09 | 4.85E-06 |
| C16orf52 | rs11640609 | 21688744 | 463.5223 | 432.2775 | 8.13E-09 | 4.85E-06 |
| C16orf52 | rs12445216 | 21690559 | 463.5223 | 432.2775 | 8.13E-09 | 4.85E-06 |
| FEZ1 | rs10893404 | 125472616 | 485.9629 | 462.2588 | 3.98E-07 | 0.000119 |
| FEZ1 | rs17140116 | 125488404 | 485.9629 | 462.2588 | 3.98E-07 | 0.000119 |
| FEZ1 | rs3731459 | 125516064 | 485.9629 | 462.2588 | 3.98E-07 | 0.000119 |
| FEZ1 | rs11220159 | 125483171 | 485.9629 | 462.2588 | 3.98E-07 | 0.000119 |
| BAI2 | rs12035217 | 32212514 | 414.7965 | 391.9195 | 6.11E-07 | 0.00014 |
| BAI2 | rs12037019 | 32213655 | 414.7965 | 391.9195 | 6.11E-07 | 0.00014 |
| BAI2 | rs12040661 | 32209650 | 414.7965 | 391.9195 | 6.11E-07 | 0.00014 |
| FEZ1 | rs7106795 | 125560180 | 485.1514 | 463.149 | 9.62E-07 | 0.000205 |
| LRRC20 | rs3957144 | 71740691 | 449.4964 | 428.681 | 1.78E-06 | 0.000355 |
| RSPH9 | rs943084 | 43770150 | 537.6922 | 517.6295 | 2.64E-06 | 0.000483 |
| SOX7 | rs17708276 | 10199548 | 544.3079 | 524.4454 | 2.93E-06 | 0.000483 |
| MT1E | rs4784693 | 56631559 | 581.5737 | 561.721 | 2.94E-06 | 0.000483 |
| GINS2 | rs2581287 | 86362552 | 577.3715 | 557.6022 | 3.07E-06 | 0.000483 |
| HSF5 | rs12450714 | 55676536 | 485.3592 | 467.3885 | 7.86E-06 | 0.001174 |
| NT5E | rs9362215 | 86136433 | 271.8001 | 254.0323 | 8.74E-06 | 0.001243 |
| BFSP1 | rs6044410 | 16812202 | 485.9956 | 468.366 | 9.40E-06 | 0.001263 |
| CTAGE7P | rs359069 | 132150299 | 580.6306 | 563.0672 | 9.73E-06 | 0.001263 |
| LYPD3 | rs2571174 | 44934489 | 441.2252 | 423.9704 | 1.14E-05 | 0.001423 |
| C5orf55 | rs16867926 | 959224 | 435.6252 | 418.5034 | 1.23E-05 | 0.001465 |
| GSPT2 | rs5945596 | 50999987 | 85.39786 | 68.48769 | 1.37E-05 | 0.001527 |
| KCNH8 | rs13090964 | 19818481 | 585.7376 | 568.842 | 1.38E-05 | 0.001527 |
| PEX11G | rs7507526 | 8553322 | 447.951 | 431.631 | 1.87E-05 | 0.001965 |
| HJURP | rs13390788 | 235624835 | 455.2194 | 438.9885 | 1.96E-05 | 0.001965 |
| CCL23 | rs7211421 | 33451680 | 628.6232 | 612.4093 | 1.97E-05 | 0.001965 |
| EDAR | rs260674 | 109599256 | 437.5376 | 421.8906 | 2.66E-05 | 0.002561 |
| ZNF45 | rs2571174 | 44934489 | 202.7557 | 187.48 | 3.23E-05 | 0.002962 |
| NFIA | rs2365465 | 62133112 | 251.7482 | 236.4964 | 3.27E-05 | 0.002962 |
| C5orf55 | rs10058912 | 948117 | 433.3027 | 418.1983 | 3.54E-05 | 0.003054 |
| EDAR | rs6716645 | 109622445 | 437.5215 | 422.4393 | 3.58E-05 | 0.003054 |
| GINS2 | rs2696838 | 86366001 | 581.827 | 566.8889 | 3.86E-05 | 0.003203 |
| METTL11A | rs10819503 | 132093021 | 161.8524 | 147.1112 | 4.28E-05 | 0.00328 |
| METTL11A | rs10988323 | 132093466 | 161.8524 | 147.1112 | 4.28E-05 | 0.00328 |
| METTL11A | rs10988334 | 132105533 | 161.8524 | 147.1112 | 4.28E-05 | 0.00328 |
| MFSD4 | rs823149 | 205735229 | 481.3271 | 466.6713 | 4.48E-05 | 0.003345 |
| ZNF225 | rs2571174 | 44934489 | 196.1947 | 181.7012 | 4.88E-05 | 0.003555 |
| TSPAN6 | rs2097516 | 100222288 | 375.544 | 361.1095 | 5.04E-05 | 0.00358 |
| C5orf55 | rs920980 | 896983 | 433.7698 | 419.3833 | 5.17E-05 | 0.003587 |
| CXorf57 | rs5962575 | 104981504 | 447.2619 | 433.4485 | 6.99E-05 | 0.004744 |
| FOXD2 | rs12760654 | 47669325 | 424.7335 | 411.0106 | 7.33E-05 | 0.004866 |
| WHAMM | rs17158811 | 84248466 | 34.851 | 21.18241 | 7.55E-05 | 0.004866 |
| IPW | rs7496436 | 24648891 | 431.3491 | 417.7084 | 7.66E-05 | 0.004866 |
| RAB6C | rs3094411 | 130380525 | 477.6244 | 464.1332 | 8.29E-05 | 0.00505 |
| BEND4 | rs16854020 | 42117559 | 529.026 | 515.5424 | 8.32E-05 | 0.00505 |
| KIAA0664L3 | rs17708876 | 31398466 | 472.5035 | 459.0499 | 8.46E-05 | 0.00505 |
| EDAR | rs6749207 | 109607256 | 439.5024 | 426.241 | 9.36E-05 | 0.0053 |

AIC.rd = Akaike Information Criterion for the reduced model i.e., a model without genotype-age interaction term; AIC.full = Akaike Information Criterion for the full model i.e., a model with genotype-age interaction term; Adjusted_pvalue = p-value corrected for multiple testing.

# Table S5: The top 50 genes with heritable transcriptional activity; the best fit model for these genes was AE (A=additive genetic effect, E=unique environmental effect)

| Gene symbol | Gene name | Best fit model | Heritability estimate |
| --- | --- | --- | --- |
| MDGA1 | MAM domain containing glycosylphosphatidylinositol anchor 1 | AE | 0.989 |
| FAM110C | family with sequence similarity 110, member C | AE | 0.980 |
| HLA-DRB5 | major histocompatibility complex, class II, DR beta 5 | AE | 0.977 |
| HLA-DQB1 | major histocompatibility complex, class II, DQ beta 1 | AE | 0.976 |
| FAM118A | family with sequence similarity 118, member A | AE | 0.971 |
| PPP1R17 | protein phosphatase 1, regulatory subunit 17 | AE | 0.968 |
| HLA-DQA2 | major histocompatibility complex, class II, DQ alpha 2 | AE | 0.968 |
| LOC338817 | uncharacterized LOC338817 | AE | 0.964 |
| LOC253039 | uncharacterized LOC253039 | AE | 0.960 |
| ZNF630 | zinc finger protein 630 | AE | 0.958 |
| GAS1 | growth arrest-specific 1 | AE | 0.956 |
| ERAP2 | endoplasmic reticulum aminopeptidase 2 | AE | 0.951 |
| BEGAIN | brain-enriched guanylate kinase-associated | AE | 0.951 |
| LRRC37A2 | leucine rich repeat containing 37, member A2 | AE | 0.950 |
| SNORA70 | small nucleolar RNA, H/ACA box 70 | AE | 0.948 |
| LILRA3 | leukocyte immunoglobulin-like receptor, subfamily A (without TM domain), member 3 | AE | 0.948 |
| NNAT | Neuronatin | AE | 0.948 |
| CHRNA7 | cholinergic receptor, nicotinic, alpha 7 (neuronal) | AE | 0.947 |
| PAWR | PRKC, apoptosis, WT1, regulator | AE | 0.945 |
| ADAMTS8 | ADAM metallopeptidase with thrombospondin type 1 motif, 8 | AE | 0.944 |
| GSTM1 | glutathione S-transferase mu 1 | AE | 0.944 |
| MYCBPAP | MYCBP associated protein | AE | 0.944 |
| LOC100288778 | WAS protein family homolog 1 pseudogene | AE | 0.943 |
| LOC254099 | uncharacterized LOC254099 | AE | 0.942 |
| CNTNAP2 | contactin associated protein-like 2 | AE | 0.942 |
| SPATA20 | spermatogenesis associated 20 | AE | 0.941 |
| CYP2B7P1 | cytochrome P450, family 2, subfamily B, polypeptide 7 pseudogene 1 | AE | 0.940 |
| LOC650623 | BEN domain containing 3 pseudogene | AE | 0.939 |
| USP32P1 | ubiquitin specific peptidase 32 pseudogene 1 | AE | 0.939 |
| RGS17 | regulator of G-protein signaling 17 | AE | 0.938 |
| HCG4 | HLA complex group 4 (non-protein coding) | AE | 0.936 |
| TCL1B | T-cell leukemia/lymphoma 1B | AE | 0.936 |
| BTN3A2 | butyrophilin, subfamily 3, member A2 | AE | 0.935 |
| LOC654433 | uncharacterized LOC654433 | AE | 0.934 |
| NIPAL4 | NIPA-like domain containing 4 | AE | 0.934 |
| MTUS2 | microtubule associated tumor suppressor candidate 2 | AE | 0.934 |
| TREML4 | triggering receptor expressed on myeloid cells-like 4 | AE | 0.934 |
| APOBEC3B | apolipoprotein B mRNA editing enzyme, catalytic polypeptide-like 3B | AE | 0.933 |
| DCLK2 | doublecortin-like kinase 2 | AE | 0.932 |
| C17orf97 | chromosome 17 open reading frame 97 | AE | 0.931 |
| TEKT4P2 | tektin 4 pseudogene 2 | AE | 0.931 |
| KANSL1-AS1 | KANSL1 antisense RNA 1 | AE | 0.931 |
| CCDC163P | coiled-coil domain containing 163, pseudogene | AE | 0.930 |
| SIGLEC14 | sialic acid binding Ig-like lectin 14 | AE | 0.930 |
| LOC100134868 | uncharacterized LOC100134868 | AE | 0.929 |
| PGM5 | phosphoglucomutase 5 | AE | 0.927 |
| LOC283089 | hypothetical LOC283089 | AE | 0.927 |
| DPYSL4 | dihydropyrimidinase-like 4 | AE | 0.927 |
| COL9A3 | collagen, type IX, alpha 3 | AE | 0.926 |
| LOC339666 | uncharacterized LOC339666 | AE | 0.925 |

# Table S6: List of 80 candidate genes and the TaqMan assays used for validation

| Gene symbol | Gene name | TaqMan assay ID |
| --- | --- | --- |
| APOL4 | apolipoprotein L, 4 | Hs00540930_m1 |
| ETV7 | ets variant 7 | Hs00903229_m1 |
| C1QC | complement component 1, q subcomponent, C chain | Hs00757779_m1 |
| C8G | complement component 8, gamma polypeptide | Hs00167188_m1 |
| HP | Haptoglobin | Hs00605928_g1 |
| NGFR | nerve growth factor receptor | Hs00609977_m1 |
| SMTNL1 | smoothelin-like 1 | Hs00418171_m1 |
| MMP23B | matrix metallopeptidase 23B | Hs04187882_g1 |
| IFNG | interferon, gamma | Hs00989291_m1 |
| SEPT4 | septin 4 | Hs00910208_g1 |
| SATB2 | SATB homeobox 2 | Hs00392652_m1 |
| FZD4 | frizzled family receptor 4 | Hs00201853_m1 |
| ASGR2 | asialoglycoprotein receptor 2 | Hs00154160_m1 |
| RNASE3 | ribonuclease, RNase A family, 3 | Hs01923184_s1 |
| MDK | midkine (neurite growth-promoting factor 2) | Hs00171064_m1 |
| DDB2 | damage-specific DNA binding protein 2, 48kDa | Hs03044953_m1 |
| BLVRA | biliverdin reductase A | Hs00167599_m1 |
| TCN1 | transcobalamin I (vitamin B12 binding protein, R binder family) | Hs01055542_m1 |
| SPHK1 | sphingosine kinase 1 | Hs00184211_m1 |
| LMCD1 | LIM and cysteine-rich domains 1 | Hs00205871_m1 |
| LAP3 | leucine aminopeptidase 3 | Hs00429769_m1 |
| C1QA | complement component 1, q subcomponent, A chain | Hs00706358_s1 |
| IGFBP2 | insulin-like growth factor binding protein 2, 36kDa | Hs01040719_m1 |
| CLEC3B | C-type lectin domain family 3, member B | Hs00162844_m1 |
| SARNP | SAP domain containing ribonucleoprotein | Hs00793492_m1 |
| PRTN3 | proteinase 3 | Hs01597752_m1 |
| NAALADL1 | N-acetylated alpha-linked acidic dipeptidase-like 1 | Hs00193839_m1 |
| AMH | anti-Mullerian hormone | Hs00174915_m1 |
| CTSL | cathepsin L | Hs00377632_m1 |
| ZSCAN9 | zinc finger and SCAN domain containing 9 | Hs00196838_m1 |
| PRSS23 | protease, serine, 23 | Hs00359912_m1 |
| NT5C3B | 5'-nucleotidase, cytosolic IIIB | Hs00369454_m1 |
| MARCO | macrophage receptor with collagenous structure | Hs00198935_m1 |
| CCL23 | chemokine (C-C motif) ligand 23 | Hs00270756_m1 |
| CXCL10 | chemokine (C-X-C motif) ligand 10 | Hs01124251_g1 |
| TBKBP1 | TBK1 binding protein 1 | Hs00207037_m1 |
| CTSK | cathepsin K | Hs00166156_m1 |
| CTSG | cathepsin G | Hs00175195_m1 |
| LXN | Latexin | Hs00220138_m1 |
| ELF3 | E74-like factor 3 (ets domain transcription factor, epithelial-specific ) | Hs00963881_m1 |
| FEZ1 | fasciculation and elongation protein zeta 1 (zygin I) | Hs00192714_m1 |
| SFRP5 | secreted frizzled-related protein 5 | Hs00169366_m1 |
| BATF2 | basic leucine zipper transcription factor, ATF-like 2 | Hs00293780_m1 |
| ETS1 | v-ets avian erythroblastosis virus E26 oncogene homolog 1 | Hs00428293_m1 |
| SATB1 | SATB homeobox 1 | Hs00161515_m1 |
| SPTBN1 | spectrin, beta, non-erythrocytic 1 | Hs00162271_m1 |
| GZMH | granzyme H (cathepsin G-like 2, protein h-CCPX) | Hs00277212_m1 |
| PLAG1 | pleiomorphic adenoma gene 1 | Hs00231236_m1 |
| ZNF229 | zinc finger protein 229 | Hs00970997_m1 |
| HIVEP2 | human immunodeficiency virus type I enhancer binding protein 2 | Hs00198801_m1 |
| AXIN2 | axin 2 | Hs00610344_m1 |
| LRRN3 | leucine rich repeat neuronal 3 | Hs01087516_m1 |
| ZNF154 | zinc finger protein 154 | Hs01068902_m1 |
| ZNF135 | zinc finger protein 135 | Hs00987427_g1 |
| TSPYL5 | TSPY-like 5 | Hs00603217_s1 |
| PRKCA | protein kinase C, alpha | Hs00925193_m1 |
| MAN1C1 | mannosidase, alpha, class 1C, member 1 | Hs00220595_m1 |
| PDGFRB | platelet-derived growth factor receptor, beta polypeptide | Hs01019589_m1 |
| ZNF551 | zinc finger protein 551 | Hs00292939_m1 |
| ANK3 | ankyrin 3, node of Ranvier (ankyrin G) | Hs00253210_m1 |
| SERPINB10 | serpin peptidase inhibitor, clade B (ovalbumin), member 10 | Hs00192370_m1 |
| RAPGEF3 | Rap guanine nucleotide exchange factor (GEF) 3 | Hs00183449_m1 |
| RNASE4 | ribonuclease, RNase A family, 4 | Hs00377763_m1 |
| DYNLT1 | dynein, light chain, Tctex-type 1 | Hs00831821_s1 |
| MAPK8IP1 | mitogen-activated protein kinase 8 interacting protein 1 | Hs00271363_m1 |
| ELANE | elastase, neutrophil expressed | Hs00975994_g1 |
| CYB5R2 | cytochrome b5 reductase 2 | Hs00212055_m1 |
| CAPG | capping protein (actin filament), gelsolin-like | Hs00156249_m1 |
| GSTM4 | glutathione S-transferase mu 4 | Hs00426432_m1 |
| NUDT18 | nudix (nucleoside diphosphate linked moiety X)-type motif 18 | Hs00368715_m1 |
| CCL4 | chemokine (C-C motif) ligand 4 | Hs00237011_m1 |
| NRG1 | neuregulin 1 | Hs00247620_m1 |
| PLA2G16 | phospholipase A2, group XVI | Hs00912734_m1 |
| ATF3 | activating transcription factor 3 | Hs00231069_m1 |
| NR1H3 | nuclear receptor subfamily 1, group H, member 3 | Hs00172885_m1 |
| ARG1 | arginase 1 | Hs00968979_m1 |
| PPP1R3G | protein phosphatase 1, regulatory subunit 3G | Hs03805942_s1 |
| FAM109B | family with sequence similarity 109, member B | Hs03025388_s1 |
| MDP1 | magnesium-dependent phosphatase 1 | Hs04194678_s1 |
| HOXB7 | homeobox B7 | Hs00270131_m1 |
